# A Modified LBP Operator-Based Optimized Fuzzy Art Map Medical Image Retrieval System for Disease Diagnosis and Prediction

**DOI:** 10.3390/biomedicines10102438

**Published:** 2022-09-29

**Authors:** Anitha K., Radhika S., Kavitha C., Wen-Cheng Lai, S. R. Srividhya, Naresh K.

**Affiliations:** 1 Department of Computing Technologies, School of Computing, College of Engineering and Technology, SRM Institute of Science and Technology, SRM Nagar, Kattankulathur, Chennai 603203, India; 2 Department of Computer Science and Engineering, Saveetha School of Engineering, Saveetha Institute of Medical and Technical Sciences, Chennai 602107, India; 3 Department of Computer Science and Engineering, Sathyabama Institute of Science and Technology, Chennai 600119, India; 4 Bachelor Program in Industrial Projects, National Yunlin University of Science and Technology, Douliu 640301, Taiwan; 5 Department Electronic Engineering, National Yunlin University of Science and Technology, Douliu 640301, Taiwan; 6 School of Computer Science and Engineering, Vellore Institute of Technology, Vellore 632014, India

**Keywords:** LBP variants, image retrieval, feature indexing, FAM classifiers, DEFAMNet

## Abstract

Medical records generated in hospitals are treasures for academic research and future references. Medical Image Retrieval (MIR) Systems contribute significantly to locating the relevant records required for a particular diagnosis, analysis, and treatment. An efficient classifier and effective indexing technique are required for the storage and retrieval of medical images. In this paper, a retrieval framework is formulated by adopting a modified Local Binary Pattern feature (AvN-LBP) for indexing and an optimized Fuzzy Art Map (FAM) for classifying and searching medical images. The proposed indexing method extracts LBP considering information from neighborhood pixels and is robust to background noise. The FAM network is optimized using the Differential Evaluation (DE) algorithm (DEFAMNet) with a modified mutation operation to minimize the size of the network without compromising the classification accuracy. The performance of the proposed DEFAMNet is compared with that of other classifiers and descriptors; the classification accuracy of the proposed AvN-LBP operator with DEFAMNet is higher. The experimental results on three benchmark medical image datasets provide evidence that the proposed framework classifies the medical images faster and more efficiently with lesser computational cost.

## 1. Introduction

### 1.1. Motivation

**Figure 1:**
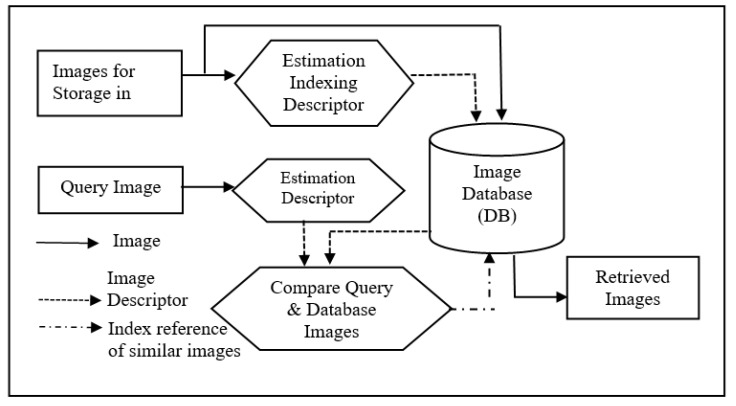
General Architecture of CBIR System.

Advanced developments in computer hardware, storage technology, and the availability of intelligent algorithms have provided opportunities for maintaining records, data, and information in digital form. The information available in the stored data leads to faster development, improvement, detection, forecasting, analysis, education, and invasions. The challenge at hand is to frame a methodology that can search and retrieve the required information from these huge digital databases. An overview of a Retrieval System architecture is depicted in Figure 1. Keywords relevant to requirement information are presented as queries to a search process. The records in the database are indexed using their related keywords. The searching process looks for matches in the index of the database to the query and displays the best matches along with locations of records. Queries for the retrieval system, indexing, and the search algorithm adopted for classification and matching measures play a vital role in deciding the performance.

Health care is one among various applications of image retrieval systems. The improvements and availability of advanced technologies and methods of digital storage have created a broad scope for adopting Electronic Medical Record (EMR) systems. EMR systems contain information about the health of an individual [1]. The information in the medical records includes the patient’s personal details, administrative data, allergies, vital symptoms, medicinal histories, lab test results, radiology images, diagnoses, medications, progress details, and follow-up dates. One/many of the pieces of information in the record shall have a significant feature suitable to be the index for the record and key for representing the record in the EMR system. 

The need is to frame an efficient retrieval system that can extract features to index, search, and retrieve the records relevant to queries on large image databases. The challenge is to develop a well-structured system that is on par with techniques like human annotation. MIR Systems provide a prominent scope for developing and improving the services in the field of medicine. Many MIR systems have been developed and are available. In this paper, we propose an MIR System adopting LBP-based indexing and a FAM-based classifier with an efficient retrieval performance.

The main contributions in the proposed are outlined below:

⮚ The framework adopts a novel LBP operator, namely, AvN-LBP.
⮚ The proposed descriptor addresses the issues due to noisy images and includes the information available in the neighborhood pixels.
⮚ An optimized FAM network is modeled as a Classifier.
⮚ The Deferential Evolution Algorithm (DE) is used for evolving the FAM network, namely, DEFAM.
⮚ A MIR System, namely, DEFAMNet, to retrieve the required images from medical databases, is developed, trained, and tested using benchmark databases.
⮚ A modified Mutation operator is implemented during the evolution of the FAM Network.

### 1.2. Related Work

Visual patterns in images are better represented by Texture. LBP is broadly adopted by researchers in image classification studies such as face detection, industrial applications, and pattern recognition. The Texture-based LBP descriptor [2] is an efficient and simple operator derived from a pre-defined threshold segmentation method. The neighborhood pixels are compared with a threshold and represented as ‘0’ and ‘1’ and finally converted into binary numbers. The computational simplicity and robustness of the LBP operator to variations in illumination is an undebatable choice for various practical applications. Many enhanced and modified variants of LBP operators have been developed to overcome the challenges faced by operators and fit different applications. Modifications are incorporated either by including the features of other operators or implementing new mathematical processes for estimating the LBP decimal. 

Research articles have provided enough significance to the effectiveness of LBP operators and their variants. An enhanced LBP operator, EF-ALBP [3], by combining the local binary pattern with an edge smoothing filter and accumulation function suppresses noise, preserves edges, and extracts the margins of medical images. Experiments were performed on medical images comprising MRI, CT, and X-ray. The results demonstrate the strength and robustness of the EF-ALBP compared to other descriptors. A novel operator, namely, Local Binary Patterns Clustering (LC) is used for dermoscopic image segmentation [4]. The operator improves the segmentation process by extracting border lines, geometric details, and target regions. The performance of LC was found to be better compared to 26 popular segmentation algorithms. Color information with the LBP feature is combined to generate a maximal multi-channel LBP (MMLBP) [5] operator. The new MMLBP proved efficient in identifying similarities between images and improves the performance of the CBIR system. The size of the MMLBP operator is smaller and performed consistently across different datasets. The Attractive Center-Symmetric LBP (ACS-LBP) and Hessian matrix are combined to develop a hybrid operator Hess-ACP-LBP [6]. The new LBP operator has texture analysis capabilities and differential texture information. A similar feature with the combined capabilities of LBP and affine-invariant detectors [7] proved its ability to identify key points and descriptions from MRI images to detect mild cognitive impairment convertible. A wavelet-based LBP for classifying X-ray images using a random forest classifier [8] exhibited improved performance. Local and global features represent images and are informative to differentiate objects. A combined feature of the LBP operator and Chebyshev moments [9] is introduced and tested to classify images in the most challenging datasets of Outex, ALOT. The results were found to be very encouraging and supportive of the superiority of the LBP operator. 

Textures patterns from biomedical images [10] are extracted and the weight aspect is added to form a Multi-scale Gabor rotation-invariant local binary pattern (MGRLBP). Using the MGRLBP operator significantly improved the classification capability and reduced the false classification ratio. To include the strength of local information available in the pixels, an LBP operator accounting center-symmetric (CSLBP) [11] is developed. The operator was tested on databases of different types of images including medical images from the OASIS dataset and was found to be effective. The algorithm to estimate CSLBP features is simple and needs less computation time. The length of the operator is also considerably smaller, and the performance is on par with other operators. The influence of noise on the LBP operator makes it less suitable for facial expression recognition. An LBP operator with reduced noise in its feature extraction estimated using new neighborhoods obtained by means of pixels in radial directions and adaptive windows was derived and successfully tested [12]. A Hybrid operator combining CLBP and MSCLBP has been developed to perform classification tasks in GeoEye imagery [13] and found suitable. LBP operators and variants are based on greyscale images. Hence, information is lost during conversion from color images to greyscale images. A color-based LBP extracted directly from color images [14] is used for the classification of color images and is found to be more efficient. An LBP operator with a color difference sign and color difference is fused to form a new multiple-channel LBP (MCLBP) [15] and has the potential to perform well in color image classification. 

Experiments performed on images in well-known datasets demonstrate the superior performance of descriptors. QuLBP [16], the quantum Local Binary Pattern, has been formulated and tested using MRI and grayscale images. Extensive experimentation results were recorded, and analysis provided encouraging results of the QuLBP approach. A combination of Local Binary Pattern in three orthogonal planes (LBP-TOP) and a histogram of orientation gradients (HOG-TOP) has been used to learn brain tumors [17]. The results of testing on BRATS 2013 certify the suitability of the proposed framework in detecting brain tumors. The LBP operator in combination with the local information analysis component produced a high classification accuracy of around 99.86% in the identification of the biometrics of coronary angiography images [18]. Identifying a more suitable set of LBP variants to represent images assures the best performance of classification systems. Experiments have been performed to explore the capacity of several LBP-based descriptors to represent images. It was analyzed and recorded that LPB operators generated with the same radius and number of neighborhoods best represent the images of images [19]. HIS images have a huge amount of spatial and spectral information. Existing feature descriptors incur large computational costs. Classical LBP operators are exclusively implemented for spatial texture representation. LBP operators used to index HSI images have limitations on spatial–spectral information. To overcome the limitation, Clifford algebra-based multidimensional LBP (MDLBP) for HSI is proposed [20]. The descriptor is able to extract spatial–spectral feature from multiple dimensions. The test results of HSI classification using MDLBP outperforms the LBP variants. A new DR_LBP [21] approach including the information available in neighborhood pixels was framed and implemented for face recognition tasks and produced good results. 

An LBP operator occupies a very larger space in texture classification requirements because of its capability to represent the texture features of an image. The operator has few limitations if attended and shall be a more powerful tool for applications in more sensible areas such as security, medical, defense, etc. The three major shortfalls of an LBP operator are (1) its performance being limited by the quality of texture images because of low-resolution imaging sensors due to size constraints in transmission networks and transmission loss, (2) the robustness of the center pixel, and (3) the corruption of uniform patterns by the presence of noise.

The retrieval of records that are similar to the requirements from the database involves classifying, searching, matching, and locating the records. This complete sequence is performed by algorithms, namely, classifiers. There are several effective algorithms that are available and used as classifiers. Among the algorithms, Neural Networks with deep learning techniques have proven their suitability for Medical Image Classification (MIC). CNN architectures such as Alexnet, ResNet, Inception, Densenet, etc. are used in many of MIC studies. The medical image classification research work published is mentioned with complete analysis [22]. The survey provides a detailed insight into the experimentation and methodologies used on medical image databases for feature extraction and various classification techniques.

A Deep Convolutional Neural Network (DCNN) [23] has been trained and implemented for detecting Alzheimer’s disease in MRI brain images. DCNN has delivered better performance and enhanced precision in retrieval. CNN architectures VGG16 and InceptionV3, capsule network training, and Space Vector Machine classifiers were tested for the evaluation of their performance to classify pneumonia from chest X-ray images [24]. The results depicted showed that CNN architectures are more effective and delivered useful performance. CNN and its best-performing frameworks have been discussed in detail [25]. The discussion covers the contents of understanding medical imaging tasks such as classification, segmentation, localization, and detection. The detailed survey provided clarity about CNN in medical image processing. The tasks include analysis and alignments in the breast, chest, lungs, brain, and other organs. CNN is also implemented for various other applications such as object detection and segmentation in medical image studies [26]. The algorithms are found to be suitable for processing ultrasound images, endoscopic images, CT, PET, and MRI. CNN was trained to detect the skin lesion from the image and for measuring the border irregularity [27]. The network achieved outstanding performance. A deep-learning CNN network was employed to diagnose infections from X-ray images [28]. The analysis was performed by extracting LBP features from the images. The results were found to be very encouraging compared to results obtained in other studies. The BreastUNet [29] framework with CNN with a capability to graft features has been developed and trained to analyze mitotic nuclei in breast histopathology images. GLocal Pyramid Pattern, a variant of LBP, is used for texture recognition in Breast Cancer datasets. The framework is found suitable for successfully classifying images with mitotic nuclei and non-mitotic nuclei. Detecting various types of cells in and around the tumor matrix is very significant and supports classifying the tumor micro-environment for cancer prognostication and research. The availability of a real-time dataset plays a vital role and encourages researchers to develop and test algorithms for efficient classification and retrieval networks. A large and diverse dataset of nucleus boundary annotations and class labels with key findings during a challenging task with the dataset MoNuSAC2020 has been published [30]. The dataset has over 46,000 nuclei from 37 hospitals, 71 patients, 4 organs, and 4 nucleus types. A detailed analysis has been performed by implementing six different algorithms for classification tasks [31]. The evaluation of retrieval and classification algorithms dealt with textured 3D objects. The results and insight in the work have provided much-required contents to the research community. The images received from sensors and transmitted from remote locations will face a loss of information. The lost information may cause a variety of transformations simultaneously, such as non-rigid deformations (changes in pose), topological noise, and missing parts—a combination of nuisance factors that renders the retrieval process extremely challenging. A total of 15 retrieval algorithms were evaluated in the contest [32] which provide the details of the dataset and present comparisons among the methods. Intuitionistic fuzzy sets (IFSs) are a competitive tool during decision-making during classification tasks and resolving ambiguity and vagueness cases. A novel similarity-distance technique [33] with a better performance rating has been framed. A comparative analysis is presented to showcase the advantages of the novel similarity-distance over similar existing approaches. Furthermore, the applications of the novel similarity-distance technique in various decision-making situations have been explored. Predicting patient survival by the degree of accuracy and efficiency is the goal of any retrieval approach. An approach is demonstrated [34] to understand the importance of using classification and FS algorithms to obtain the best results faster, as it is a crucial factor in a patient’s survival. After conducting experiments and analyzing results obtained in terms of error rate and accuracy, it was concluded that the classification algorithm produces better results without combining them with the FSFA. The rich literature provides strong evidence for using an LBP operator for indexing and a Neural Network-based framework for classification. 

From the literature, a few characteristics of images, such as overlapping classes, noisy images, the sequence of presenting images for training, etc., influence performance and cause category proliferation problems in Neural Networks. This importantly influences the prediction accuracy of the classifier, and the proposed work aims to improve the accuracy. 

## 2. Materials and Methods

The proposed framework performs image indexing using a novel LBP operator, namely, AvN-LBP. The proposed descriptor addresses the issues caused due to noisy images by including the information available in the neighborhood pixels. The difficulties faced by stand-alone classification algorithms are addressed by including neural network-based classifier FAMs. To improve the computation efficiency and classification accuracy, the FAM network is optimized. DE is used for evolving a FAM network, namely, DEFAM. A MIRS framework DEFAMNet to retrieve the required images from benchmark medical image databases by using AVN-FAM for indexing and DEFAM for classification and retrieval is developed, trained, and tested.

### 2.1. Introduction to LBP Methodology

An LBP modeled by Ojala [35,36] is a descriptor used to represent texture images and is estimated at each pixel of an image *I* of size N × M. X_r,n_ denotes the (n + 1)th pixel at a distance r around the center pixel x_0,0_. The image represented by *I* in Equation (1) is of size 5 × 5 with r = 2 and, for example, X_2,4_ represents the 5th (fifth) pixel in the 2nd (second) row of image *I*.

(1) $$I_{5\times 5}=\left[\begin{array}{ccccc} x_{2,6} & x_{2,5} & x_{2,4} & x_{2,3} & x_{2,2}\\ x_{2,7} & x_{1,3} & x_{1,2} & x_{1,1} & x_{2,1}\\ x_{2,8} & x_{1,4} & x_{00} & x_{1,0} & x_{2,0}\\ x_{2,9} & x_{1,5} & x_{1,6} & x_{1,7} & x_{2,15}\\ x_{2,10} & x_{2,11} & x_{2,12} & x_{2,13} & x_{2,14} \end{array}\right]$$

The LBP descriptor is a string of binary bits 0 (zero) or 1 (one) based on a mathematical relationship between a particular pixel and the pixels present in its neighborhood. The estimation of LBP is defined in Equation (2).

(2) $$LBP_{p,r}=\sum_{n=0}^{p-1}S(x_{r,n}-x_{0,0})2^{n}$$

where

$$S(x)=\left\{\begin{array}{c} 1x\geq 1\\ 0x<1 \end{array}\right.$$

**Figure 2:**
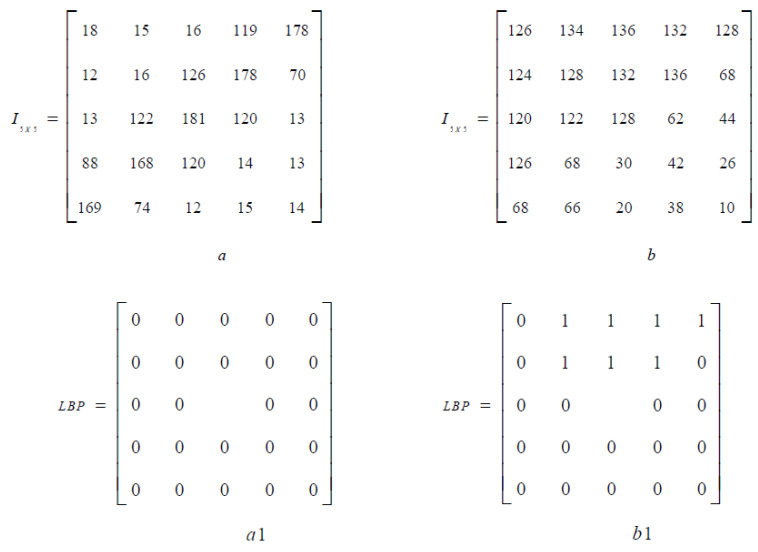
Image patches (**a**,**b**) and corresponding LBP patterns (**a1**,**b1**).

Two examples of image patches (a,b) and corresponding *LBP* factors (a1, b1) are shown in Figure 2.

Equation (3) provides the equation to compute the *h*(*k*) of length *K* = 2^*p*^, *LBP* descriptor for the complete image.

(3) $$h(k)=\sum_{i=0}^{N}\sum_{j=0}^{M}\delta (LBP_{p,r}(i,j)-k)$$

where 0≤k≤K−1; $LBP_{p,r}(i,j)$ is the *LBP* pattern of each pixel i,j of an image *I* of size NXM. 

LBP has many promising features that makes it an eminent choice for the representation of texture images. The features include invariance, simple calculation that reduces computational time, fewer assumptions and parameters, rotation invariance, and better discrimination power. However, LBP suffers from a few weaknesses that need to be addressed for adopting the descriptor for applications expecting superior performances. From Equation (3), it can be noticed that the length of the histogram depends on P and stretched to a length of ${K=2}^{p}$. Larger P values produce longer histograms. It is also evident from the process of estimating the descriptor that the presence of noise influences the LBP operators. The presence of noise in the images will produce non-uniform patterns. The performance of the stand-alone classifiers is affected because of these non-uniform patterns. To overcome the weaknesses of the LBP descriptor, the below session presents a modified LBP-based descriptor AvN-LBP for texture image representation.

### 2.2. Proposed Approach

The proposed approach is modeled explicitly to include the combined information of the central pixel and the pixels in its neighborhood and hence possesses the strength of maintaining information coming from neighbors and minimizes the effects of noise. It produces consistent patterns and is discriminative and robust to noise. The AvN-LBP operator of an image is estimated using Equation (4).

(4) $$AvN-LBP_{p,r}=\sum_{n=0}^{p-1}S(x_{{}_{r,n}}^{\prime }-\mu_{r})2^{n}$$

where

$$S(x)=\left\{\begin{array}{c} 1,x\geq 0\\ 0,x<0 \end{array}\right.$$

$$\mu_{r}=\frac{1}{p}\sum_{n=0}^{p-1}x_{r,n}^{\prime }$$

**Figure 3:**
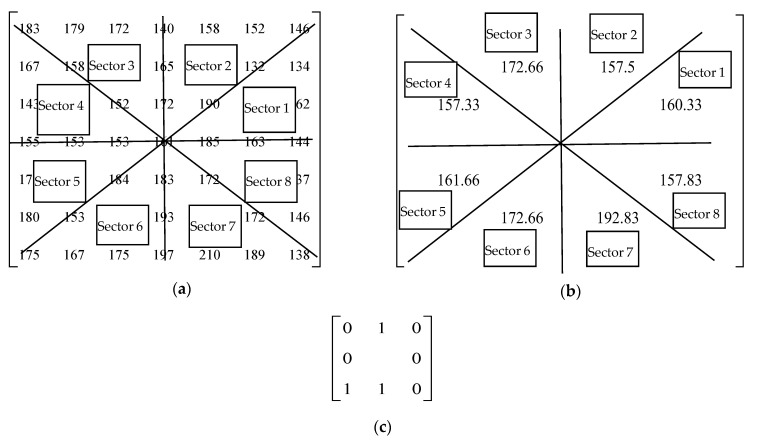
Steps detailed for the calculation of the proposed AvP-LBP descriptor for the center pixel in an example image patch. (**a**) Image Patch and sectors for R = 3, p = 8, and $\theta =45^{0}$. (**b**) Average value of the circular sector = sum (185,163,144,190,132,146)/6 = 160.33. (**c**) AvN-LBP pattern is 01100100 for $\mu_{r}$ = sum (160.33, 157.5, 172.66, 157.33, 161.66, 172.66, 192.83, 157.83)/8 =166.26 and AvN-LBP = 100.

**Figure 4:**
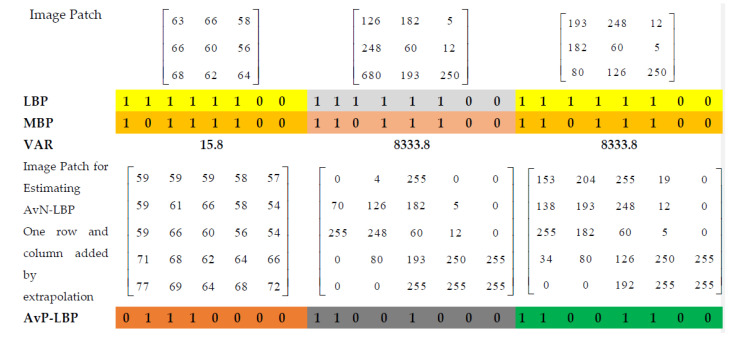
Three texture patterns and LBPs, AvN-LBPs. MBPs, and VAR values for the texture patterns.

**Figure 5:**
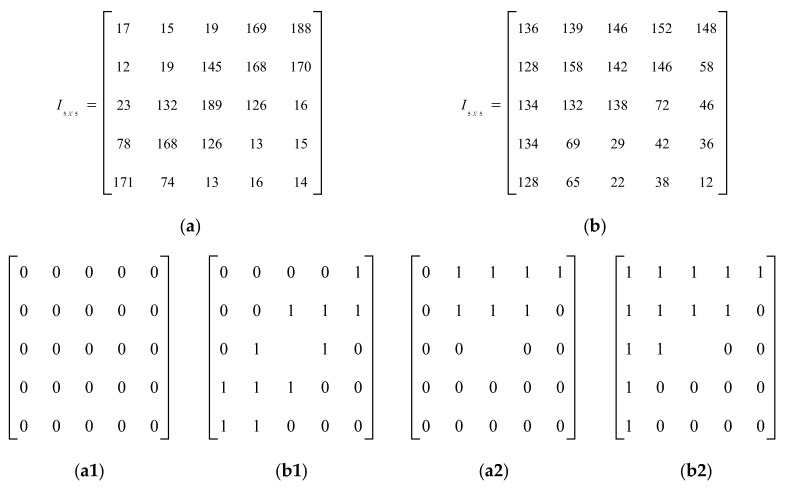
Example for effective edge preservation by proposed AvN-LBP. (**a**,**b**) Two image patches of size 5 × 5. (**a1**,**b1**) AvN-LBP preserves weak edge patterns. Two 5 × 5 example image patches are shown in (**a**,**b**). (**a1**,**a2**) are the patterns given by LBP for (**a**,**b**), respectively. (**b1**,**b2**) AvN-LBP patterns of (**a**,**b**), respectively.

**Figure 6:**
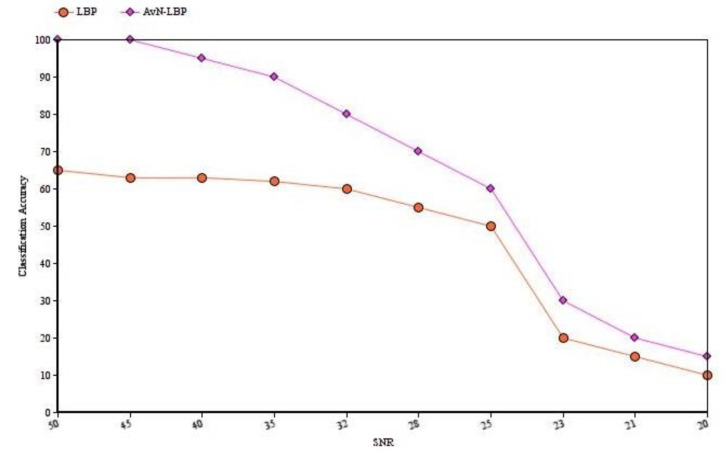
Comparison of the robustness of the proposed AvN-LBP descriptor to noise and the comparison with conventional LBP.

The $x_{{}_{r,n}}^{\prime }$ is computed by averaging the pixels in an image patch lying on an angular sector of a circle with a radius R = 2.3 and $\theta =15^{0},30^{0},45^{0}$. If $\theta =45^{0}$, the pixels in the circle with radius R from the center pixel are divided into eight sectors. The example in Figure 3 provides the steps for the calculation of AvN-LBP. The proposed AvN-LBP descriptor has three major advantages:

- Thresholding at $\mu_{r}$ tends to make local neighborhood vectors almost zero-mean. Hence, the descriptor proposed is not affected by grayscale changes and is resistant to lighting effects.
- Since the threshold is estimated from the neighborhood pixels, the pattern is more discriminative compared to LBP. Figure 4 shows the discriminative strength of the proposed descriptor.
- Weak edges are better preserved by AvN-LBP. Analyzing Figure 5, it can be noticed that the LBP patterns are not reflecting the actual distribution of pixels compared to the proposed descriptor.
- Less influenced by the presence of noise. Figure 6 provides a comparison between the signal-to-noise ratio (SNR) and the classification accuracy for the proposed AvN-LBP and conventional LBP. The results provide the evidence for the robustness of the proposed descriptor for different Gaussian noise levels added to images.

### 2.3. Fuzzy ARTMAP Architectures

#### 2.3.1. Fuzzy ARTMAP (FAM)

**Figure 7:**
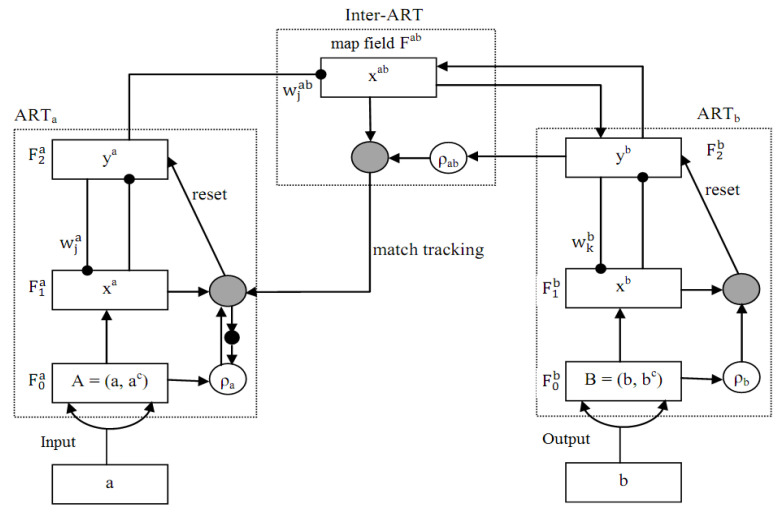
FAM Architecture.

The FAM Architecture in Figure 7 has ART modules $ART^{a}$ and $ART^{b}$. These two modules are interconnected by a mapping field. The unlabeled descriptors of images from a class are submitted to input (a) of module $ART^{a}$, and the labeled descriptor of the same class is given to input (b) of $ART^{b}$. The map field relates the descriptors to their labeled counterparts. In FAM, learning ensures that each category is connected to one node representing a particular class in the database. The classes of all categories are identified and labeled with their corresponding classes during the learning processes. Each set of patterns (a, b) given as an input to the FAM classifier during the learning process shall belong to correctly labeled classes.

In case the input pattern to $ART^{a}$ does not choose a class associated with the one presented to $ART^{b}$, FAM performs a reset operation laterally, and the vigilance parameter is varied. Thus, the network selects a different suitable category. The categories in $ART^{a}$ and $ART^{b}$ that are not committed with patterns will not have associations in the map field during the initial stages of the learning process. The focus of the work is classification learning. The images are classified into their respective classes. The output nodes F_2_ of FAM will have 10 nodes to represent a database with 10 classes. A FAM network is trained to identify the classes in a database and used as a classifier in Content-Based Image Retrieval (CBIR) systems to organize and search the images in the database based on their features. Images with the same features are considered similar and grouped as the same class. Algorithm 1 provides the algorithm for training FAM network. After proper training, a FAM network is capable of identifying the class of query images submitted to the CBIR system.



**Algorithm 1: Training FAM network**

FN_T_ = Training (F, l)//FN_T_-Trained FAM Network//F: Feature Vector, l: Label → Class of the feature vector.Initialize ρ, NE = 0//ρ = Vigilance Parameter, NE: Count training epochsWhile NE < number of training epochsI_i_ = F = (a_1_,a_2_,…,a_d_);//d: dimension of feature vectorAI_1_ = (a_1_, a_2_,…,a_d_,1-a_1_,1-a_2_,…,1-a_d_)if AI = first input of label lW = AIW←lelse for (all j) compute T_j_(AI) = $\left|AI\wedge W_{j}\right|/\alpha +\left|W_{j}\right|$J = argmax (T_j_(AI); J: Winner nodeif $\left|AI\wedge W_{j}\right|/\left|AI\right|\geq \rho$; vigilance testif l = J:$W_{k}^{new}=W_{k}^{old}+\beta (AI\wedge W_{k}^{old})$else:$\rho =MF_{k}(AI)+\in$While (more winner nodes are available)While (more training patterns are available)End



Based on the sequence of input images during the training phase of the classifier, the presence of similar characteristics between images (overlapping classes) across different classes in the dataset and the presence of noise are major reasons that increase the size of FAM networks. The problem is mentioned as a category proliferation problem [36]. The problem influences the precision of image categorization and also increases the computational cost.

#### 2.3.2. Non-Proliferation Fuzzy ARTMAP (NPFAM)

NPFAM is a FAM architecture with a modified learning process that addresses the category proliferation problem. The NPFAM framework is adopted for image retrieval [37] with an inter-ART reset model to restrict the growth of the number of categories that result in larger networks leading to category proliferation. The model decides the requirement of the newly created category and skips its creation if found not required. In addition, to ensure classification accuracy, an offline learning model is included, and the requirement of the newly created category is ensured before deciding on the deletion of a particular category. Probabilistic data for connected one/many relationships are stored in $w_{jk}^{ab}$, and the probability of an inter-ART reset during prediction is stored in weight $v_{jk}^{ab}$. These weights define the entropy (coverage) of the training set. 

#### 2.3.3. Differential Evolution of FAM Networks (DEFAM)

DEFAM [38] is an optimized FAM architecture used for image retrieval. The architecture is created by repeatedly applying DE operators to the initial population of trained FAM networks. The initial population is generated by training the FAM networks for image classification following all the reasons that cause the category proliferation problem. Due to the category proliferation problem, large networks are created. A network evolved from these populations of trained networks using DE will be able to deliver high precision classification and smaller classifier network. In the proposed work, the complex problem of optimizing the FAM network is achieved by adopting an advanced version of the DE algorithm that uses neighborhood mutation and opposition-based learning [39] for solving the complex problem of optimizing the FAM network. The DE algorithm searches in the space of solutions with the guidance of the difference between individuals. The basic phenomenon is to generate a mutation vector by differentiating and scaling two vectors of a population and adding them to the third vector in the same population. The mutation vector is crossed with the parent vector with a predefined probability to generate a target vector. Then, the most efficient vectors among the target and parent vectors are identified using a fitness function, which proceeds to the next generation. The basics of the adopted DEFAM evolution process, including a brief explanation about the proposed mutation strategy, are outlined below.

##### Initialization

Each of the trained FAM networks is represented by D dimensional vectors of size M. Similarly, N networks are considered as the initial population. Each individual vector shall be expressed as in Equation (5).

(5) $$x_{i}(G)=\left\{\begin{array}{cccc} x_{i1}, & x_{i2}, & x_{i3},\ldots \ldots , & x_{iD} \end{array}\right\}$$

where G represents the generation and i = 1 to N. 

##### Mutation

Mutation operation generates mutation vector $V_{i,G}$ corresponding to each $x_{i,G}$ target vector of the present population employing mutation strategies. Equations (6)–(9) provide the most commonly used mutation strategies

(6) $$DE/rand/1^{}\;V_{i,G}=x_{r1,G}+F\cdot (x_{r2,G}-x_{r3,G})$$

(7) $$DE/best/1^{}\;V_{i,G}=x_{best,G}+F\cdot (x_{r1,G}-x_{r2,G})$$

(8) $$DE/rand-to-best/1^{}\;V_{i,G}=x_{i,G}+F\cdot (x_{best,G}-x_{i,G})+F\cdot (x_{r1,G}-x_{r2,G})$$

(9) $$DE/rand/2^{}\;V_{i,G}=x_{r1,G}+F\cdot (x_{r2,G}-x_{r3,G})+F\cdot (x_{r4,G}-x_{r5,G})$$

where $r_{1},r_{2},r_{3,}r_{4}\&r_{5}$ are randomly selected integer numbers between (1, M), F is the scaling factor, and $x_{best,G}$ is the individual vector evaluated with the best fitness in the current generation.

DE and Particle Swarm Algorithms face difficulties to balance local and global search abilities. During the evolution process, the local search leads to premature convergence and on the other side, depending on the global search, degrades the exploring ability and increases the convergence time. Neighborhood mutation modules [39,40] provide promising results and avoid the convergence of the DE algorithm to the local optimum. Influenced by the capability and results of neighborhood approaches, a modified version of the strategy in Equation (8) based on the neighborhood approach is framed as in Equation (10).

(10) $$DE/neigh-to-best/1^{}\;V_{i}^{G}=x_{r3}^{G}+F_{1}\cdot (x_{best}^{G}-x_{r3}^{G})+F_{2}\cdot (x_{r1}^{G}-x_{r2}^{G})$$

The proposed strategy in Equation (10) avoids premature convergence, restricts incompetent mutation operators, involves individual vectors with high fitness values to reach convergence faster, and avoids large-scale global convergence. Algorithm 2 depicts the steps involved in proposed mutation technique.



**Algorithm 2: Steps for Mutation**

Input = $x_{}^{G}$for all $x_{}^{G}$ calculate fitness value using Equation (13)Sort $x_{}^{G}$ based on its Fitness valueSelect $x_{best}^{G}$ and 30% to 60% of top ranked $x_{}^{G}$Implement Mutation operation based on Equation (10)End



##### Crossover

A new test vector $U_{i,G}=(u_{1,G},u_{2,G},\ldots \ldots ,u_{i,G})$ is generated during the process by employing a binomial crossover between the target vectors $x_{i,G}$ and the mutation vector $v_{i,G}$. The procedure is defined in Equation (11)

(11) $$u_{i,G}=\left\{\begin{array}{l} v_{i,G},if\;rand_{j}(0,1)>CR{)}^{}or^{}(j=j_{rand},j=1,2,3,\ldots \ldots ,D)\\ x_{i,G},otherwise \end{array}\right\}$$

where CR is the crossover rate. CR∈[0,1] and $j_{rand}\in [1,D]$.

##### Selection

Selection is performed between the target vectors $x_{i,G}$ and test vectors $U_{i,G}$ based on their fitness. The selected vectors shall be the part of the population used for evolving the network during the next generation. Selection is performed by evaluating the fitness of $x_{i,G}$ and $U_{i,G}$ using the function f(p). The selection operation is performed following Equation (12).

(12) $$X_{i,G+1}=\left\{\begin{array}{l} U_{i,G},if\;f(U_{i,G})>f(X_{i,G})\\ x_{i,G},otherwise \end{array}\right\}$$

The fitness function for optimizing the FAM classifier is expressed in Equation (13).

(13) $$F(p)=\frac{\frac{100}{cat_{\min}}-\frac{p_{cc}(p)}{N_{a}(p)}}{\left(cat_{\max}-N_{a}(p))p_{cc}^{2}(p\right)}$$

Subjected to:

(14) $$N_{c}\leq cat_{\min}\leq cat_{\max}$$

(15) $$cat_{\min}<N_{a}(p)\leq cat_{\max}$$

where $cat_{\min}$ and $cat_{\max}$ are the minimum and maximum number of categories that can be allowed. These values shall be decided by the user. A best $cat_{\min}$ value shall be equal to the number of classes in the dataset. The fitness function in Equation (3) is derived such that DE has to evolve a FAM-based classifier such that F(p) as a minimum. An optimum FAM network is one that has a smaller number of categories $N_{a}(p)$ near to the minimum number of categories $cat_{\min}$ and delivers the highest possible accuracy $p_{cc}(p)$ equal to or nearer to 100, that is, the numerator of the fitness function in Equation (13) $\frac{100}{cat_{\min}}-\frac{p_{cc}(p)}{N_{a}(p)}$ shall be near to zero. Similarly, the denominator of the fitness function $\left(cat_{\max}-N_{a}(p))p_{cc}^{2}(p\right)$ must be very larger. Algorithm 3 portrays the steps involved in evolution of FAM network using proposed DE algorithm.



**Algorithm 3: Evolution of FAM Network**

Pop_best_fit = DE(FN_T_)Initialize β = 1.0 and CR = 0.7Set Generation Count t = 0(max = G)Create the initial population C(t) = NPWhile (stopping criteria not true)For each target x_i(_(G)ϵC(G)Create the Mutant Vector V_i_(G)Create the test vector U_i_(G)For all iEvaluate F(x_i_(G)) and F(U_i_(G))If (f(x_i_(t) ≥ f(U_i_(t))x_i_(t + 1) = x_0_(t)else xi(t + 1) = U_0_(t)Return population with best fitnessEnd



## 3. Results

The parameters for the AvN-LBP extraction, training, and optimization of the FAM network adopted during the experimentation of the proposed DEFAMNet-based MIR are provided in Table 1. The parameters used influenced the overall performance of the proposed framework. The parameters used in the proposed DEFAMNet can be grouped into three categories. The parameters related to AvN-LBP belong to the first category and decide the effectiveness of indexing in the proposed framework. p is the number of circle parts. In the proposed work, the pixels in the circle of pixels are portioned into eight at an angle of 45°. A low number of partitions will not consider the actual neighbors of the pixel, and a large number of partitions will increase computations and consume more time. Hence, an optimum value p = 8 is chosen. R is the radius of the circle considered for averaging. R = 3 means three pixels in the grid will be averaged. The radius also influences the number of computations and decides the time consumption. The second category of parameters is related to the FAM Network. The FAM dynamics are decided by a learning parameter $\beta \in \left[0,\;1\right]$, Vigilance parameter ρ $\in \left[0,1\right]$ and choice parameter α > 0. For fast learning, β = 1. We performed experimentations using different β values and decided to assign β= 0.8 during the learning process of the FAM. Vigilance parameter ρ decides the size of hyper boxes representing the category of a class in the database. ρ ≅ 1 allows larger boxes; ρ ≅ 0 restricts the size of the boxes and increases the size of the FAM network. In our work, we decided to have ρ ≅ 1, have smaller networks, and use DE for achieving the required performance. Small values of α reduce recoding during learning, hence α = 0.001 is assigned during the experimentation. DE has been adopted to optimize plenty of problems, and from the literature, β = 1 and CR = 0.7 are considered.

The proposed DEFAMNet classifier is trained for 100 epochs, with a batch size of 64, assigning a learning rate of 0.001 and training data from a medical image dataset. 

During the development of the MIR framework 40%, 30%, and 30% of the images in each class in the database are adopted for training, testing, and validation, respectively [41,42]. An NVIDIA DGX station with a processor 2.2 GHz, Intel Xeon E5-2698 (20-Core), NVIDIA Tesla V100 4 × 16 GB GPU was used.

### 3.1. Database

There are several databases available to support research on medical images. Images in each database have different features and offer interesting challenges. A MIR system shall be capable of performing in a similar pattern for any type of image. The capability of the proposed DEFAMNet medical image retrieval system is examined using the Public Lung Image Database (I-ELCAP) [43], the Open Access Series of Imaging Studies–Medical Image Resonance (OASIS–MRI) [44], and Interstitial Lung Disease (ILD) [45].

### 3.2. Evaluation of Proposed DEFAMnet Medical Image Retrieval System

The effectiveness of any retrieval system should guarantee that the retrieved images should be closer in similarity with the query images. The Average Retrieval Precision (ARP) and Average Retrieval Rate (ARR) are evaluation metrics commonly used to measure the performance of CBIR systems. The proposed DEFAMNet classifier is evaluated and analyzed by the ARP and ARR values calculated using Equations (16)–(19).

(16) $$\left|Precision\right|=P(I_{q})=\frac{N_{R}\cap N_{RT}}{n_{RT}}$$

(17) $$\left|Recall\right|=R(I_{q})=\frac{N_{R}\cap N_{RT}}{n_{R}}$$

(18) $${\left.ARP=\frac{1}{DB}\sum_{n=1}^{DB}P(I_{n})\right|}_{n\leq 10}$$

(19) $${\left.ARR=\frac{1}{DB}\sum_{n=1}^{DB}R(I_{n})\right|}_{n\geq 10}$$

where $I_{n}$ is the query image, and DB is the number of images in the database. $N_{R}$ is the number of images in the database that are similar images to the query image, $N_{RT}$ is the total number of retrieved images similar to the query image, and $N_{R}\cap N_{RT}$ provides the number of images in common between similar images in the database and similar images retrieved. $n_{R}$ is the total of the relevant images present in the database relevant to the query image, $n_{RT}$ is the total of the images retrieved by the MIR System. The results of the proposed AvN-LBP descriptor are compared with texture descriptors such as LBP [46], LTP [47], LDP [48], and other patterns [49,50].

### 3.3. Performance on the I-ELCAP Database

**Figure 8:**
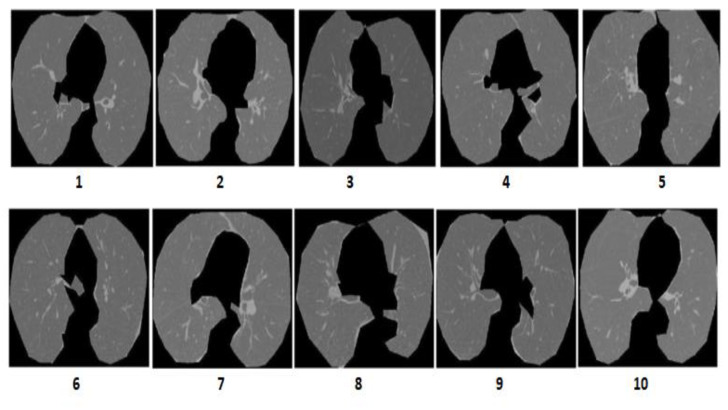
Sample images from 10 different classes of the I-ELCAP image database.

The proposed DEFAMNet framework is evaluated using the I-ELCAP dataset. The I-ELCAP dataset has been used because of its popularity in evaluating MIR systems. The dataset has 10 classes with 100 images in each class. The weights associated with DEFAMNet are trained with 10% of the images from each class of the dataset. The proposed DEFAMNet is tested with the remaining images. One sample image from each of the 10 classes in the I-ELCAP database are shown in Figure 8. A comparison of performance in terms of APR and ARR achieved by DEFAMNet and other approaches along with a performance comparison between AvN-LBP with other LBP variants are presented in Table 2 and Table 3. DEFAMNet retrieved 100% similar images for the query image from eight classes and an average performance of 99.8% for all 10 classes. It can also be noticed from Table 2 and Table 3 that the best performance among other techniques considered for comparison is 99.1%.

### 3.4. Retrieval Analysis on the OASIS–MRI Database

**Figure 9:**
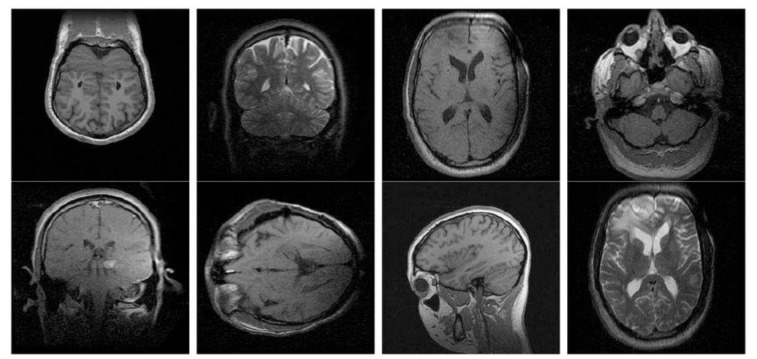
Sample images from different classes of the OASIS database.

Experimentations are performed on the publicly available OASIS–MRI medical image database to evaluate AvN-LBP and DEFAMNet. OASIS comprises 421 images. The images belong to patients aged between 18 and 96. For the sample images in the OASIS database, the images are organized into four classes. Each class consists of 124, 102, 89, and 106. Figure 9 presents one sample image from each of the four classes. Briefly, 10% of the images from the database are used to tune the weights of DEFANET and balance, and 90% of the images are used to evaluate DEFAMNet’s retrieval performance. The results achieved by AvN-LBP and DEFAMNet are compared with existing approaches and variants of LBP on the OASIS dataset and are summarized in Table 4. It is clear from the results; the best performance of existing approaches is 84.3%, and that of the proposed DEFAMNet is 93.01% in terms of average retrieval accuracy.

### 3.5. Result Analysis on the ILD Database

**Figure 10:**
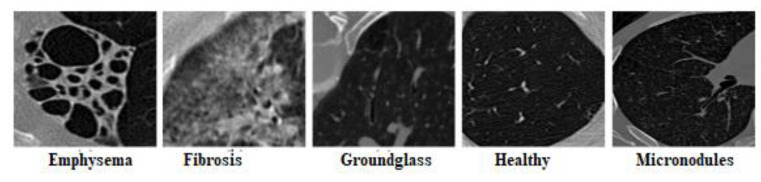
Sample images from five classes of ILD database.

In actuality, the medical records are a combination of images and clinical data. The evaluation of the proposed framework is performed on the ILD database. The ILD database has valuable content and contains 658 images of interstitial lung disease along with clinical data of patients for research studies. One sample image from five classes is provided in Figure 10. The classes of ILD are micronodules (173 images), ground glass (106 images), emphysema (53 images), fibrosis (187 images), and healthy (139 images). The DEFAMNet weights are tuned with 10% of the images and tested with the remaining 90% of the images. The performance of the proposed approach in terms of ARP compared with other approaches and feature descriptors on the ILD dataset is given in Table 5. DEFAMNet performed with 96.06% average retrieval accuracy against the 92.4% best performance of the approaches considered for comparison.

From the experimental results on the three databases used for evaluation, the proposed DEFAMNet and AvN-LBP outperformed the existing approaches used for medical image retrieval and prove that the proposed perform with better results across different types of image datasets and different classes in the dataset.

### 3.6. MIR System Adopting NPFAM and DEFAMNet Classifiers

The medical images in the OASIS database are categorized using two classifiers, namely, NPFAM and DEFAMNet. The classifiers are chosen for comparison because both are FAM networks and address the category proliferation problem. On the other hand, the procedure adopted to construct the classification model is entirely different. This section compares the performance of these classifiers in medical image classification. The classifiers trained with AvN-LBP operators produced better results. Training was undertaken with 30 percent of the images (36, 30, 26, and 32) from each of the four classes. The trained classifiers were evaluated by testing to classify 421 images in the database (124, 102, 89, and 106). After training, the network was tested to classify the images from the training set, and we found that DEFAMNet with AvN-LBP performed at 100%. The trained network was then tested to classify the balance of 40% of images (50, 40, 35, and 43) from each class. Table 6 provides the comparison of accuracy considering only the top 25 retrieved images based on similarity.

### 3.7. Retrieval Accuracy Obtained on Different Body Parts

Medical images of different parts of the human body exhibit different texture patterns. Hence, the performance of the DEFAMNet MIR system is examined with diverse X-Ray images of body parts. Medical images from the internet are collected for six different parts (classes) of the body, namely, the chest, head, foot, neck, palm, and spine [54,55,56]. A total of 150 images has been used, with 25 images in each class. The classifier is trained with 10 images per class and tested with the remaining images. Table 7 shows the retrieval accuracy achieved by the system with only AvN-LBP and AvN-LBP with classifiers.

## 4. Discussion

Retrieval time, required resources, and retrieval performance are factors considered to evaluate the suitability of retrieval systems for performing the retrieval task. The execution time consists of the time required for feature extraction from query images and retrieval (classification, searching, and retrieving similar images from the database). The system’s time consumption depends on the procedure for the extraction of the feature descriptor. The larger the operator, the longer it will take to extract the vector and retrieve the images from the database. The retrieval time is also affected by the organization of databases and the use of classifiers in the retrieval system. Table 8 summarizes the time required for the estimation of the feature descriptor and retrieval time in seconds over the OASIS–MRI database using the proposed AvN-LBP and other descriptors considered for comparison.

**Figure 11:**
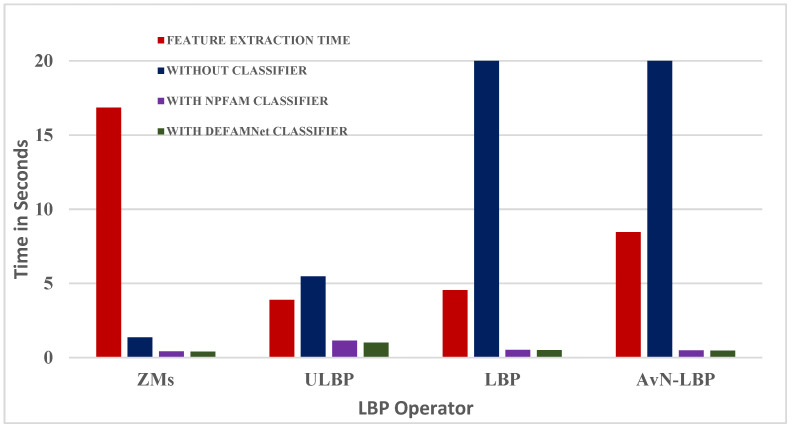
Comparison of total CPU Elapsed time and Retrieval time in seconds for LBP, ULBP, LDEP, ZMs, and the proposed LBP/VAR-based feature descriptors over OASIS-MRI databases.

The extraction time of images from the OASIS database with the proposed method is less than that of LDEP and ZMs, but greater than that of LBP and ULBP. Table 8 and Figure 11 provide the comparison of the total CPU time (feature extraction time + retrieval time) required for image retrieval using the proposed method and other feature descriptors on the OASIS–MRI database. The results show that the computation time of ULBP is less than that of the proposed AvN-LBP operator. The extra time is compensated for with improved retrieval accuracy.

## 5. Conclusions

This paper has proposed a local texture-based descriptor, AvN-LBP, a modified version of the well-known LBP. The proposed descriptor addresses issues due to noisy images by including the information available in the neighborhood pixels to better represent the images and avoid the loss of valuable information. In addition, a FAM classifier evolved using the DE algorithm that avoids the category proliferation problem is implemented. Modifications are performed during the mutation stage to make the DE algorithm more suitable for the intended application of MIR. The complete proposed NPFAM-based MIR system accommodating AvN-LBP for indexing medical images and DEFAMNet for retrieval is subjected to extensive experimentation, and the results presented provide the proof of its suitableness. In future, if the time consumption and resource requirements are reduced, the proposed system can be more competitive with the existing MIR systems. Experimentation is performed on smaller databases, but the size of the datasets influences the retrieval results, hence the proposed framework shall be implemented on larger datasets and needs to be extended to real-time application.

## Figures and Tables

**Table 1:** Parameters adopted for the AvN-LBP extraction, training, and optimization of the FAM network adopted during experimentation.

				MODULE					
	AvN-LBP			FAM				DE	
PARAMETER	p	R	Θ	Ρ	*β*	δ	α	β	CR
VALUE	8	3	45^0^	1.0	0.8	0.2	0.001	1.0	0.7

**Table 2:** Comparison of Percentage Retrieval Accuracy (ARP) on the I-ELCAP database.

OPERATOR/ ALGORITHM	CLASS										
	1	2	3	4	5	6	7	8	9	10	Avg
**LBP** [46]	56.6	72.7	64.7	89.5	67.8	91.6	80.4	99.7	78.4	90.7	79.1
**LTP** [47]	49.6	52.6	58.5	80.8	48.2	77.2	55.3	92.5	61.5	74.8	65.4
**GLBP** [46]	67.8	84.2	77.8	92.3	79.2	89.3	77.1	99.1	84.1	97.4	84.7
**LMeP** [49]	77.3	75.1	68.1	92.6	73.5	93.4	86.8	100	80.1	87.2	83.2
AvN-LBP *	**85.6**	**89.9**	**86.7**	**95.6**	**91.1**	**98.5**	**91.4**	**99.4**	**97.1**	**97.1**	**93.4**
**GLCM** [47]	76.8	55.1	55.1	54.9	49.9	74.2	68.5	94.7	32.3	72.4	63.3
**GLMeP** [49]	82.5	82.2	85.6	95.5	74.6	97.5	90.1	100	82.7	94.2	88.4
**ResNet** [50]	100	96.8	100	100	100	100	85.7	96.8	96.8	100	97.3
**AlexNet** [51]	82.9	99.1	95.2	78.9	96.6	92.6	99.1	73.2	93.8	62.5	84.1
**VGG-16** [52]	63.8	100	79.4	85.7	90.9	59.1	100	83.9	100	95.2	82.1
**RetrieveNet** [53]	100	100	100	98.1	100	98.1	100	100	100	94.2	99.1
DEFAMNet *	**100**	**100**	**100**	**99.4**	**100**	**100**	**100**	**100**	**100**	**98.6**	**99.8**

* Proposed Method.

**Table 3:** Comparison of Percentage Retrieval Accuracy (ARR) on the I-ELCAP database.

OPERATOR/ ALGORITHM	CLASS										
	1	2	3	4	5	6	7	8	9	10	Avg
**LBP [46]**	32.4	40.5	38.6	67.0	36.2	54.2	48.5	81.2	47.8	72.7	51.9
**LTP [47]**	23.1	25.8	23.8	52.1	20.6	38.5	22.8	40.9	26.4	40.6	31.4
**GLBP [46]**	34.5	42.5	41.5	66.0	37.7	51.1	40.2	72.1	47.4	76.7	51.0
**LMeP [49]**	37.4	35.4	28.9	73.0	37.9	52.9	53.3	95.6	43.0	69.6	53.7
AvN-LBP *	**49.8**	**51.2**	**47.9**	**72.5**	**41.1**	**52.9**	**52.7**	**76.1**	**45.6**	**84.6**	**57.5**
**GLCM [47]**	33.2	28.2	16.5	26.4	19.7	34.2	27.6	52.9	15.4	42.1	29.6
**GLMeP [49]**	38.7	38.3	37.3	71.4	36.5	62.2	56.3	89.6	44.8	70.4	54.6
**ResNet [50]**	100	100	96.7	86.7	100	100	100	100	100	90.2	97.3
**AlexNet [51]**	96.7	76.7	66.7	100	93.3	83.3	33.3	90.2	100	100	84.2
**VGG-16 [52]**	100	96.7	90.1	100	33.3	96.7	60.5	86.7	100	66.7	82.4
**RetrieveNet [53]**	100	100	100	100	100	100	96.1	96.8	100	98.5	99.1
DEFAMNet *	**100**	**97.0**	**99.5**	**99.0**	**100**	**100**	**100**	**99.0**	**100**	**98.0**	**99.3**

* Proposed Method.

**Table 4:** Comparison of Retrieval Accuracy (ARP) on the OASIS database.

OPERATOR/ ALGORITHM	CLASS				
	1	2	3	4	Avg
**LBPSEG** [45]	43.21	38.03	28.32	46.44	39.01
**LTP** [47]	56.33	36.70	34.97	50.02	45.17
**CSLBP** [45]	44.72	40.15	31.17	48.27	41.06
**GLDP** [47]	48.72	40.09	38.41	41.52	42.23
AvN-LBP *	58.91	61.38	51.3	66.43	59.51
**LDP** [47]	46.29	36.37	36.82	45.56	41.8
**LMEBP** [54]	46.17	40.17	36.83	49.17	43.08
**ResNet** [50]	78.01	57.74	73.91	86.52	75.62
**AlexNet** [51]	88.01	54.51	62.51	73.82	68.52
**VGG-16** [52]	75.74	57.14	52.41	70.74	66.15
**RetrieveNet** [53]	90.01	71.25	82.15	95.92	84.3
DEFAMNet *	96.16	84.64	91.23	100	93.01

* Proposed Method.

**Table 5:** Retrieval Accuracy (ARP) in percentage on ILD database.

OPERATOR/ ALGORITHM	CLASS					
	Emphysema	Fibrosis	Groundglass	Healthy	Micronodules	Avg
**LBP** [46]	26.79	47.85	35.66	28.71	28.79	33.53
**LTP** [47]	36.79	49.82	45.66	38.71	37.72	41.73
**LTCop** [50]	31.32	50.82	44.15	28.71	63.88	43.77
**LTrP** [54]	42.07	51.87	49.27	41.79	57.65	48.52
AvN-LBP *	56.42	65.64	57.64	48.24	65.86	58.76
**ResNet** [50]	90.98	91.49	88.96	83.78	72.49	82.86
**AlexNet** [51]	62.52	92.68	88.23	55.78	83.78	75.47
**VGG-16** [52]	50.75	79.45	56.95	73.68	94.72	75.89
**RetrieveNet** [53]	99.89	98.92	91.75	80.56	96.36	92.40
DEFAMNet *	100	97.92	94.56	92.90	99.12	96.90

* Proposed Method.

**Table 6:** Accuracy in percentage achieved on the OASIS database for the top 25 retrieved images.

DESCRIPTOR	CLASSIFIER	
	NPFAM	DEFAMNet
**LBP**	87.64	90.00
**CS-LBP**	89.41	91.76
**NI-LBP**	86.47	88.82
**LTP**	88.82	90.58
**AVN-LBP**	91.88	93.71

**Table 7:** Percentage accuracy achieved by MIR Systems on the database of human parts for the top 25 retrieved results.

CLASS	RETRIEVAL ACCURACY		
	AvN-LBP	AvN-LBP + NPFAM	AvN-LBP + DEFAM
**CHEST**	54.6	89.1	91.2
**HEAD**	66.8	86.4	90.3
**FOOT**	60.2	85.2	89.2
**NECK**	71.4	87.6	91.7
**PALM**	69.2	88.7	94.4
**SPINE**	56.1	86.2	90.3

**Table 8:** Feature extraction and retrieval time in secs over OASIS–MRI databases using proposed LBP/VAR and other descriptors considered for comparison.

Eature Descriptor	Feature Extraction Time	Retrieval Time in Seconds		
		Without Classifier	With NPFAM Classifier	With DEFAMNet Classifier
**ZMs**	16.85	1.37	0.42	0.41
**ULBP**	3.89	5.48	1.14	1.02
**LBP**	4.56	20.41	0.52	0.51
**AvN-LBP**	8.46	22.32	0.49	0.47

## Data Availability

The datasets used and/or analyzed during the current study are available from the corresponding author upon reasonable request.
